# Soft, Stretchable, High-Sensitivity, Multi-Walled Carbon Nanotube-Based Strain Sensor for Joint Healthcare

**DOI:** 10.3390/nano15050332

**Published:** 2025-02-21

**Authors:** Zechen Guo, Xiaohe Hu, Yaqiong Chen, Yanwei Ma, Fuqun Zhao, Sheng Guo

**Affiliations:** School of Mechanical, Electronic and Control Engineering, Beijing Jiaotong University, Beijing 100044, China

**Keywords:** MWCNTs, strain sensors, soft electronics, exoskeleton, healthcare

## Abstract

Exoskeletons play a crucial role in joint healthcare by providing targeted support and rehabilitation for individuals with musculoskeletal diseases. As an assistive device, the accurate monitoring of the user’s joint signals and exoskeleton status using wearable sensors is essential to ensure the efficiency of conducting complex tasks in various scenarios. However, balancing sensitivity and stretchability in wearable devices for exoskeleton applications remains a significant challenge. Here, we introduce a wearable strain sensor for detecting finger and knee joint motions. The sensor utilizes a stretchable elastic conductive network, incorporating multi-walled carbon nanotubes (MWCNTs) into Ecoflex. The concentration of MWCNTs has been meticulously optimized to achieve both a high gauge factor (GF) and stability. With its high sensitivity, the sensor is enabled to be applied in the angle monitoring of finger joints. By integrating the sensor with human knee joints and an exoskeleton device, it can simultaneously detect the flexion and extension movements in real-time. This sensor holds significant potential for enhancing exoskeleton performance and improving joint healthcare technologies.

## 1. Introduction

The exoskeleton, worn as an extension of the human body, has garnered considerable attention due to its remarkable ability to offer locomotion assistance and enhance human capabilities in the fields of both rehabilitation and biomechanics [1,2,3,4]. To ensure effective bi-directional human–exoskeleton interaction, it is imperative to exhibit a seamless human–machine interaction, which not only augments the limbs’ mobility but also guarantees a real-time interpretation and response to the user’s intentions [5]. Typically, the integration of sensor technology in this process is pivotal, as sensors are capable of discerning the user’s physical state and movement intentions, providing crucial information that enables a productive and efficient collaboration between the user and the exoskeleton system [6,7].

It is imperative to employ sensors with a high sensitivity and a broad signal monitoring range. Typically, human strain signals fall below 100% [8], while the knee flexion angle often exceeds 90° [9]. To cater to the demands of both the knee joint and the exoskeleton, there is an urgent need for strain sensors that can operate efficiently under extensive strain with the utmost sensitivity. Advancements in wearable electronics have brought forward strain sensors that are both stretchable, flexible, and highly stable, offering a promising solution for detecting human body signals for different on-body sites [10,11,12]. Based on their operational mechanisms, flexible strain sensors are mainly categorized as resistive, capacitive, piezoelectric, or triboelectric sensors [12,13,14,15]. Among these types, resistive strain sensors have garnered significant attention due to their simplistic structure, straightforward fabrication procedure, and accessible signal acquisition [16,17,18]. Nevertheless, the sensitivity of resistive strain sensors needs to be improved [19].

In an effort to boost the sensitivity of soft strain sensors, a variety of two-dimensional nanomaterials have been employed, such as graphene [20,21,22], carbon nanotubes (CNTs) [23,24,25], and so on. Owing to their intrinsic properties, these materials grant the electronic skin with exceptional capabilities of a wide range of stimuli. Among numerous two-dimensional nanomaterials, the nanoscale CNTs and multi-walled carbon nanotubes (MWCNTs) combined with an elastic substrate exhibit outstanding sensitivity and stretchability along the applied strain [26,27]. For instance, Mai et al. [17] proposed a MWCNTs–Ecoflex nanocomposite resistive sensor with conductive filler clusters that demonstrated high stretchability (~200%) with a maximum gauge factor (GF) of 0.4. To enhance the performance of the strain sensor, a highly conductive and stretchable electrospun polyurethane (TPU) fiber yarn decorated with MWNTs and SWNTs was prepared with a GF of 1.24 in the strain range of 20~100% [10]. Amjadi et al. [28] presented an ultra-soft strain sensor composed of CNT percolation network–silicone rubber nanocomposite thin films; it possessed super-stretchability (~500%) and had a GF of ~2.42. Although significant progress has been made in developing strain sensors based on CNTs and MWCNTs, achieving an optimal balance between stretchability and high sensitivity remains a significant challenge.

To tackle the challenges above, we developed a MWCNTs/Ecoflex-based strain sensor, aiming to achieve high stretchability combined with excellent sensitivity. The sensor consisted of a MWCNTs/Ecoflex nanocomposite as the sensing layer, as well as two layers of Ecoflex as the flexible substrate and packaging layer, respectively. The sensor was manufactured through a cost-effective casting procedure to form a homogeneous layered structure, minimizing the delamination caused by a mismatch of Young’s modulus between layers. The concentration of MWCNTs was optimized to achieve excellent stretchability, high sensitivity, and long-term stability. With its high sensitivity, the sensor enables applications such as the angle monitoring of finger joints and knee joints. Additionally, by integrating the sensor with an exoskeleton device, the flexion and extension movements were simultaneously detected in real-time. This innovative approach offers a promising solution for accurate and comfortable joint motion detection in exoskeletons.

## 2. Materials and Methods

### 2.1. Materials

MWCNTs were purchased from Dazhan Nano China (Guangdong) Co., Ltd., Guangdong, China, with a purity of no less than 99%, a density of approximately 0.06–0.09 g/cm^3^, an inner diameter ranging between 3 and 15 nm, and a length within the range of 15–30 μm. Ecoflex silicone (Ecoflex 0030) was purchased from Smooth-On, Macungie, PA, USA.

### 2.2. Fabrication of the Sensor

**Figure 1:**
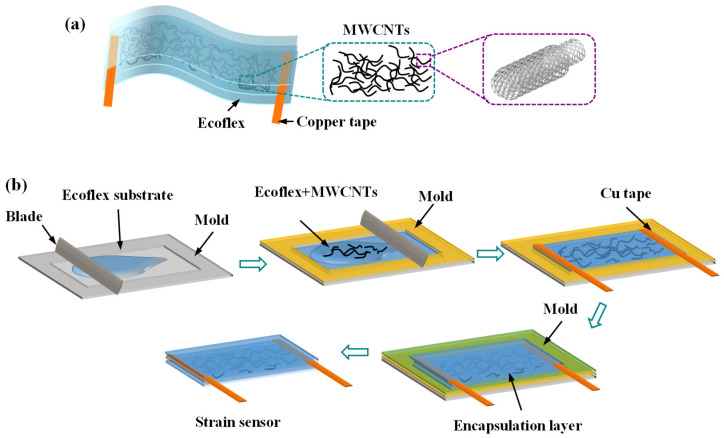
(**a**) Schematic illustration of the strain sensor and (**b**) the fabrication process.

A schematic diagram of the sensor structure is shown in Figure 1a. The sensor features a three-layer structure, with a substrate and an encapsulation layer made of Ecoflex, while the sensing layer comprises a nanoscale sensing network created by embedding MWCNTs in Ecoflex. The fabrication process of the sensor is shown in Figure 1b. Firstly, the Ecoflex component A and B precursor solutions were mixed and stirred thoroughly, followed by a vacuum application to effectively remove entrapped bubbles. The mixed Ecoflex solution was poured into a mold with a dimension of 30 × 10 × 0.1 mm^3^. To ensure the uniform distribution of the Ecoflex throughout the mold, a blade was used to spread the solution evenly. The mold was heated on a hot plate to 50 °C for 1 h to facilitate the complete curing of the Ecoflex. MWCNTs were mixed with Ecoflex at weight fractions of 6.5 wt%, 7.0 wt%, and 7.5 wt%, respectively. The mixture was stirred and then subjected to vacuum for 5 min to eliminate bubbles. Using a polyimide tape as a secondary template, the mixture of MWCNTs and Ecoflex was poured into the mold and evenly distributed using a blade. The mold was then heated on a hot plate at 50 °C for 1 h to ensure that the mixture was fully cured to form a sensing layer with a thickness of 180 μm. Using conductive silver epoxy, a copper tape was securely bonded to the sensing layer, functioning as an electrode. The curing condition for the conductive silver epoxy was set to 150 °C for 1.5 h. An encapsulation layer of Ecoflex was cast on the surface of the sensing layer, with a thickness of 80 μm. The device was delicately extracted from the mold to create the strain sensor.

### 2.3. Characterization

The morphologies of the MWCNTs, the sensing layer, and the cross-section diagrams of the sensor were observed using field-emission scanning electron microscopy (SEM, HITACHI SU8020, Tokyo, Japan). The morphology of the MWCNTs was captured using a high-resolution field-emission scanning electron microscopy (HRTEM, FEI Tecnai G2 F30, Hillsboro, OR, USA). The structural characteristics of the MWCNTs embedded in Ecoflex were measured using Raman spectroscopy (Renishaw inVia, Renishaw, Wotton-under-Edge, UK). An excitation laser source with a wavelength of 532 nm was placed on the sample to collect the Raman spectral data from 250 to 3200 cm^−1^.

### 2.4. Measurement of the Electromechanical Properties

To investigate the GF of the nanocomposite strain sensor, a custom-made in situ tensile testing setup was used. Uniaxial tensile tests were conducted using a single-axis linear guide platform driven by a linear stepping motor. The strain of the sensor varied from 0 to 105%, with a step size of 15%. In the frequency response and stability tests, the strain of the sensor was 30%. The electrical resistance of the strain sensor was measured using a data acquisition board (TS-M0002, Tushen Technology, Shanghai, China).

## 3. Results and Discussion

### 3.1. Characterization of the Sensing Layer

**Figure 2:**
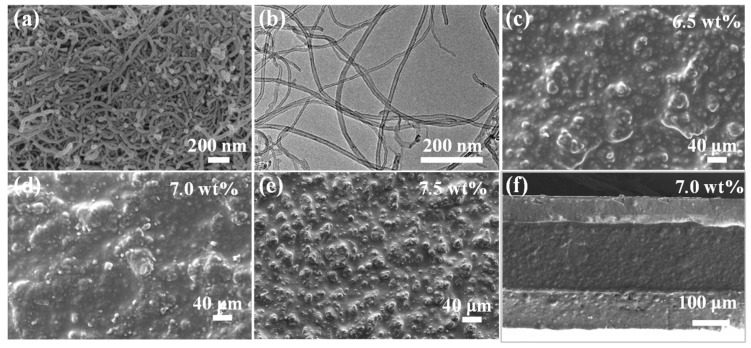
(**a**) Scanning electron microscopy (SEM) image of the multi-walled carbon nanotubes (MWCNTs) with a nanoscale diameter. (**b**) High-resolution field-emission scanning electron microscopy (HRTEM) image of MWCNTs. (**c**–**e**) SEM images of the surfaces of the sensing layer with MWCNTs weight fractions of 6.5 wt%, 7.0 wt%, and 7.5 wt%. (**f**) Cross-sectional view of the sensor with MWCNTs weight fraction of 7.0 wt%.

The morphology of the MWCNTs is presented in Figure 2a,b, exhibiting a nanoscale dimension with an approximate diameter of 15 nm. The SEM images of the sensing layer with MWCNTs embedded in Ecoflex matrix at weight fractions of 6.5 wt%, 7.0 wt%, and 7.5 wt% are displayed in Figure 2c–e. It is evident that the MWCNTs are distributed throughout the Ecoflex elastomeric matrix. Figure 2f illustrates the cross-section of the strain sensor, confirming the multilayer structure of the sensor and its sandwich configuration. It can be seen that the layers are tightly bonded together at the interfaces to form a continuous structure. The thickness of the sensor is 360 μm.

**Figure 3:**
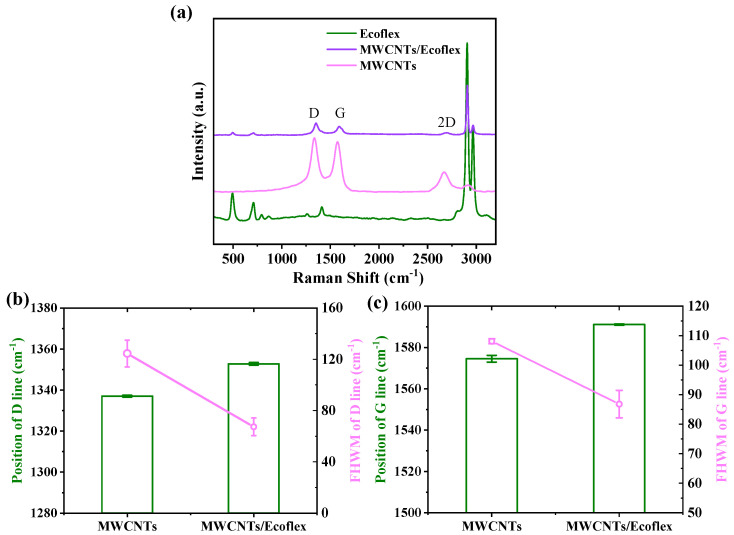
(**a**) Raman spectra of the MWCNTs embedded in Ecoflex with a weight fraction of 7.0 wt% compared with that of the Ecoflex elastomeric matrix and pristine MWCNTs. (**b**) Comparison of the position and full width at half maximum (FHWM) of the D line and (**c**) G line for MWCNTs and MWCNTs/Ecoflex.

Raman spectroscopy was used to study the structural properties of the materials. The Raman bands of Ecoflex are at 497 cm^−1^, 711 cm^−1^, and 2905 cm^−1^. The two characteristic peaks in MWCNTs/Ecoflex are recorded at ≈1352 cm^−1^ and ≈1591 cm^−1^, and can be assigned to the D band and G band of the MWCNTs, respectively (Figure 3a) [29]. The G band is attributed to the tangential stretching of the organized graphitic crystals formed by sp2 hybrid carbon atoms, while the D band corresponds to scattering caused by local defects in the carbon [30]. A 2D band at 2695 cm^−1^ arises from the stacking arrangement of the nanosheets [31]. Comparisons of the position and full width at half maximum (FHWM) of the D line and G line for pristine MWCNTs and MWCNTs/Ecoflex are shown in Figure 3b,c. The embedding of MWCNTs in Ecoflex results in changes in the D and G line position of MWCNTs and their FHWM. The intensity ratios of the disorder-induced D line and G band (*I*_D_/*I*_G_) for pristine MWCNTs and MWCNTs/Ecoflex are 0.84 and 0.85, respectively. The defect-induced D-mode relative intensity increased after the embedding of MWCNTs in Ecoflex, indicating the increased concentration of defects [32].

### 3.2. Electromechanical Performances and Mechanism of the Sensor

The electromechanical performance of the flexible strain sensor begins with an evaluation of sensitivity, which is defined by the GF [33,34]. This can be determined through the use of the following formula:

(1) $$\mathrm{GF}=\frac{\Delta R/R_{0}}{\Delta L/L_{0}}$$

where *R*_0_ and *L*_0_ are the initial resistance and length of the sensor without applied strain. ∆*R* and ∆*L* represent changes in resistance and external strain, respectively.

**Figure 4:**
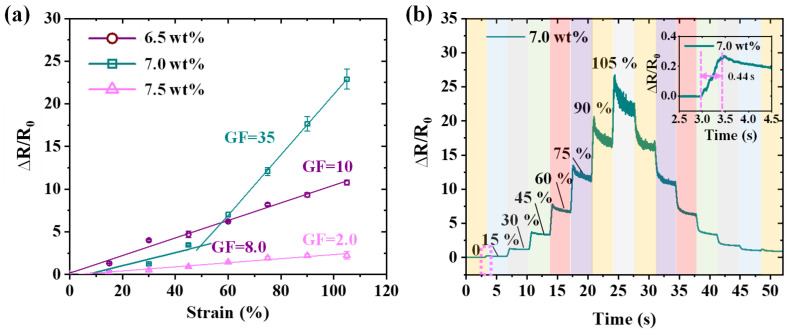
(**a**) Relative resistance variation curves as tensile strain changes within the strain range of 0 to 105% of the sensors. (**b**) Real-time ∆*R*/*R*_0_ strain curves under various tensile strains. The inset shows the real-time response upon applied 15% tensile strain.

The effects of MWCNTs concentrations (6.5 wt%, 7.0 wt%, and 7.5 wt%) on the GF of the sensor were investigated. Mechanically, the sensor was subjected to tensile strains, resulting in a corresponding change in its resistance. The GF decreases as the MWCNTs weight fraction increases in the 0 to 60% strain range, as shown in Figure 4a. However, when the strain exceeds 60%, the GF of the sensor with a 7.0 wt% weight fraction rapidly rises, achieving a GF of 35, which is significantly higher than that of other MWCNTs weight fractions. The sensor boasts a maximum tensile strain of 105%, effectively fulfilling the requirements for detecting human strain information, which is generally less than 100% [8,35]. The relative resistance variation curve as strain increases across a range from 0% to 105% is illustrated in Figure 4b. Minimal ∆*R*/*R*_0_ hysteresis is observed, indicating real-time responsiveness to strain changes. The response time of the sensor upon applied 15% tensile strain was 0.44 s (Figure 4b—inset).

**Figure 5:**
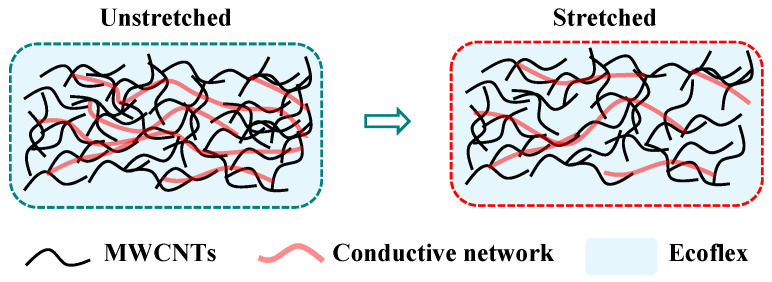
Change mechanism of the MWCNTs-embedded elastomer under applied strain.

The mechanism of the electrical conductivity of the sensor is shown in Figure 5. The MWCNTs are embedded in Ecoflex, forming percolating conductive pathways. Throughout the tensile process, the gap between the centerlines of neighboring MWCNTs is larger than an individual MWCNT’s diameter but smaller than a threshold distance. This allows electrons to traverse the polymer matrix through tunneling, forming a quantum-based conductive junction [26,36]. Nevertheless, if the exerted strain surpasses this critical threshold distance, the electrical pathway will be fully disrupted.

**Figure 6:**
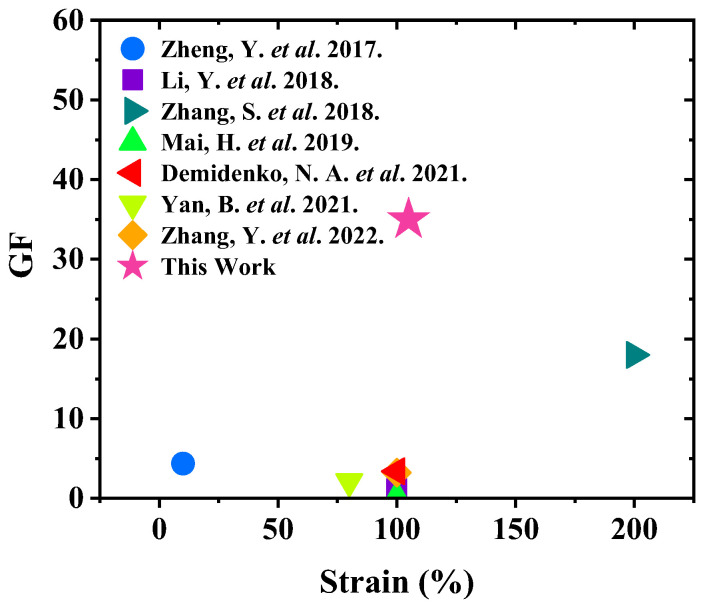
Comparison of the performance of the sensor and those reported in the literature [10,16,17,23,24,25,31].

Although many other CNT-based strain sensors have demonstrated high GFs and strain ranges [37,38], the sensor in this work is targeted at MWCNTs and Ecoflex. Therefore, Figure 6 shows a comparison of sensing performance of the GF and strain of the sensor with other reported CNTs and MWCNTs-based sensors. The strain sensor in this work exhibits a relatively higher GF in the linear strain range.

**Figure 7:**
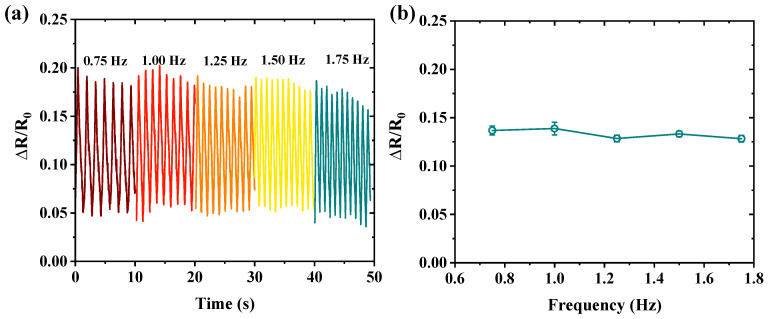
(**a**) Real-time frequency response of the strain sensor. (**b**) Relative ∆*R*/*R*_0_ variation curve at different tensile frequencies at a strain of 30%.

The stretching–relaxation response plays a crucial role in determining the sensor’s responsiveness to external strains [39]. Figure 7a presents the frequency response of the sensor under cyclic tensile tests at an applied strain of 30%, demonstrating excellent dynamic stability. Notably, the output of the sensor is less affected by the frequency variations shown in Figure 7b.

**Figure 8:**
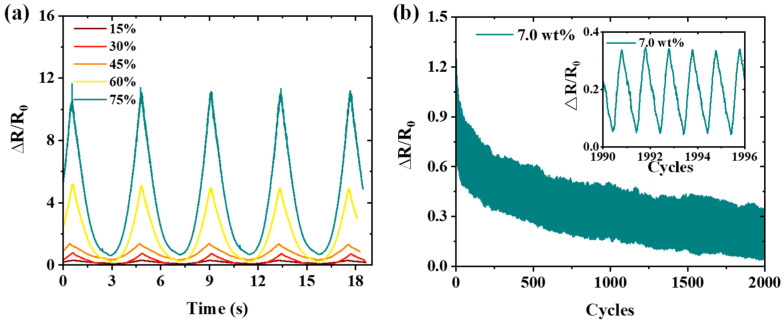
(**a**) Dynamic stretch and release cycle response of the sensor under various strains (15–75%). (**b**) Real-time fatigue resistance of the sensor at 30% strain for 2000 loading–release cycles.

The real-time resistance response of the sensor subjected to tensile strains ranging from 15% to 75% followed by relaxation demonstrates notable stability, as shown in Figure 8a. To further assess the durability and robustness, a cyclic strain of 30% was applied over 2000 cycles, as shown in Figure 8b. The ∆*R*/*R*_0_ values of the sensor demonstrated a slight downward trend within the strain cycles, which is attributed to the MWCNTs with a high aspect ratio having plenty of complicated motions, which form extra conductive networks [16]. Nevertheless, it can still be verified that the MWCNTs/Ecoflex-based sensor has a remarkable durability against cyclical loading and unloading processes in spite of the gradually decreasing phenomenon. Furthermore, the strain response curve exhibits no significant changes even under prolonged cyclic tensile tests, affirming its exceptional mechanical resilience. Upon undergoing fatigue testing, the sensor demonstrates integrity without any observable signs of structural damage, further reinforcing its durability and robustness.

### 3.3. Applications of the Sensor for Human Motion Monitoring

**Figure 9:**
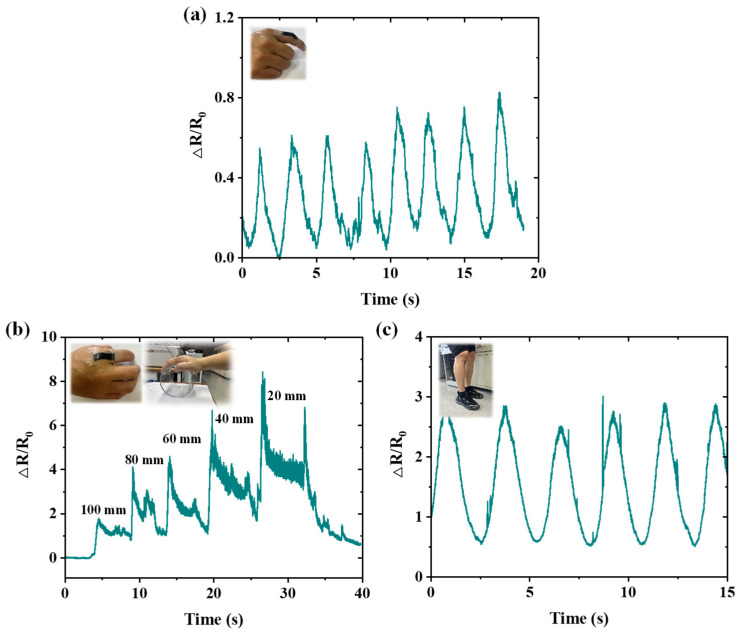
Strain sensor used for joint movement detection. (**a**) Bending and extension movement detection of the sensor on interphalangeal joints (PIPs) of the index finger. (**b**) Grasping cylinders with different diameters (20 mm, 40 mm, 60 mm, 80 mm, and 100 mm). (**c**) Detection of the bending and extension of the knee joint.

The MWCNTs/Ecoflex-based sensor presents great potential in serving as a wearable electronic due to its sensitivity, stretchability, and durability. In order to demonstrate the potential application of the strain sensor as a wearable device, the sensor is assembled into various positions to monitor different human motions. As shown in Figure 9a, when the interphalangeal joints (PIPs) of the index finger undergo a 30° flexion cycle, reciprocal signals are detected by the sensor, which indicates that the strain sensor is stable during the monitoring process.

Then, the sensor was mounted on the metacarpophalangeal joint (MP) of the index finger. The palm was controlled to grasp cylinders with diameters of 20 mm, 40 mm, 60 mm, 80 mm, and 100 mm, as shown in Figure 9b. As the diameter of the gripped cylinder decreases, the sensor’s resistance undergoes a more pronounced change due to the reduction in curvature, resulting in intensified joint deformation. The observed peaks in the curve are likely attributed to the contact between the finger and the cylinder during the grasping action.

Additionally, the sensor possesses the ability to evaluate the knee joint flexion, specifically during walking motions (Figure 9c). When the knee is bent during locomotion, the sensor stretches, resulting in an elevation of resistance. This unique characteristic is highly beneficial for accurately determining the angle formed by the calf during walking, as well as for estimating the number of steps taken based on variations in resistance. The lower limb exoskeleton serves a pivotal function in augmenting human mobility.

**Figure 10:**
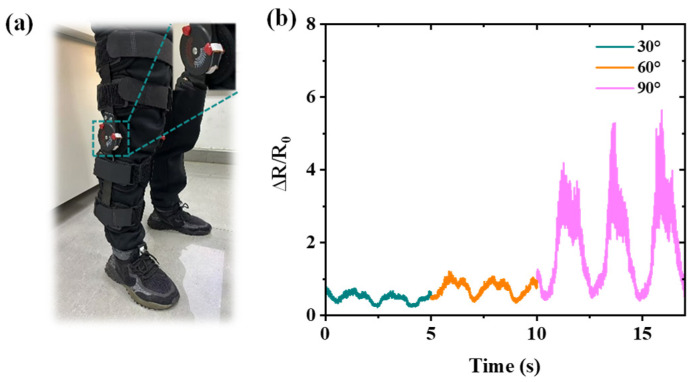
Exoskeleton movement monitoring with different bending angles. (**a**) Photograph of the sensor on an exoskeleton device. (**b**) Exoskeleton movement monitoring during flexion and extension at angles of 30°, 60°, and 90°.

In addition to detecting knee movement, the sensor can also detect the movement of an exoskeleton device (Figure 10a). The sensor was attached at the knee position of the exoskeleton under the movements of flexion and extension during various angles of 30°, 60°, and 90°, as shown in Figure 10b, which demonstrates capabilities of simultaneously detecting the flexion and extension movements of both the human knee joint and the exoskeleton device. The sensor’s compatibility with exoskeletons is evident for several reasons. Firstly, compared with other sensors based on PDMS and TPU yarns, this sensor utilizes Ecoflex as the substrate, which ensures high stretchability and an elastic modulus closely matching that of human skin, thereby minimizing mechanical energy loss [10,16]. Secondly, in comparison to existing MWCNTs/Ecoflex sensors, we have optimized the composition by increasing the proportion of MWCNTs in Ecoflex, leading to a significant enhancement in sensor performance [17]. This refinement results in a higher GF, bolstering the detection capabilities of both exoskeletons and subtle deformations in human joints. Moreover, the sensor’s overall thickness, just 360 μm, ensures exceptional skin and exoskeleton conformity. Lastly, our sensor boasts an extensive strain range, making it well suited for detecting both minor and significant strains in human joints and exoskeleton joints.

## 4. Conclusions

In conclusion, we have developed a stretchable resistive strain sensor based on a MWCNTs/Ecoflex electroactive layer. The sensor demonstrates a continuous sandwich structure, and the layers are closely connected without stratification. The concentration of the MWCNTs in Ecoflex is optimized to achieve the highest GF. Detailed electromechanical analyses elucidate the high sensitivity within a strain range of 60% to 105%, satisfying the needs of human strain information. In addition, frequency and stability tests demonstrate the stability of the sensor. With continuous finger joint movement detection, the sensor provides valuable joint motion information. The sensor’s capability to simultaneously detect the flexion and extension movements of both the human knee joint and the exoskeleton device offers the potential to optimize the performance of exoskeletons and enhance joint healthcare technologies.

## Data Availability

The original contributions presented in this study are included in the article; further inquiries can be directed to the corresponding author.
